# Clinicopathological features, clinical efficacy on 101 cases of rectal gastrointestinal stromal tumors, and the significance of neoadjuvant therapy

**DOI:** 10.1186/s12893-021-01397-8

**Published:** 2021-11-19

**Authors:** Hongxin Yang, Chaoyong Shen, Xiaonan Yin, Zhaolun Cai, Qian Wang, Bo Zhang

**Affiliations:** 1 Department of Gastrointestinal Surgery, West China Hospital, Sichuan University, Chengdu, Sichuan Province China; 2 Department of Gastrointestinal Surgery, The Affiliated Hospital of Guizhou Medical University, Guiyang, Guizhou Province China

**Keywords:** Rectal, Gastrointestinal stromal tumor, Neoadjuvant treatment, Imatinib mesylate, Disease-free survival, Overall survival

## Abstract

**Objective:**

To investigate the clinicopathological features and clinical efficacy among 101 cases of rectal gastrointestinal stromal tumors (GISTs) and to investigate the significance of imatinib mesylate (IM) neoadjuvant therapy.

**Methods:**

The clinicopathological features, treatment methods, perioperative data, and prognosis of the patients were summarized and analysed in 101 patients with rectal GISTs who received treatment in the Gastrointestinal Surgery of West China Hospital of Sichuan University and the Affiliated Hospital of Guizhou Medical University from August 2002 to November 2020 in China.

**Results:**

A total of 101 patients, including 64 males and 37 females, were aged from 22 to 79 years (55.4 ± 12.2 years). Among the 70 patients who underwent direct surgery, 8 were very low risk cases, 10 were low risk cases, 7 were intermediate risk cases, and 45 were high risk cases. Cox regression analysis showed that postoperative IM adjuvant treatment improved the disease-free survival (DFS) and overall survival (OS) of 52 intermediate and high risk patients. Among the 31 patients who received neoadjuvant therapy, the objective response rate (ORR) was 83.9% (26/31), and the disease control rate (DCR) reached 96.8% (30/31). Subgroup analysis was also conducted based on the tumour diameter. (1) Among the 36 patients with a diameter ≤ 5 cm, two patients received IM neoadjuvant therapy, while 34 patients received direct surgery. Neither univariate nor Cox regression analysis found that neoadjuvant therapy affected DFS and OS. (2) Among the 65 patients with a diameter > 5 cm, 29 received IM neoadjuvant therapy, and 36 received direct surgery. Patients who underwent neoadjuvant therapy had less blood loss (P = 0.022), shorter postoperative hospital stay (P = 0.001), increased anal retention rate (93.1% vs. 72.2%, P = 0.031), and decreased enterostomy rate (10.3% vs. 33.3%, P = 0.037) than those who underwent direct surgery. Cox regression analysis suggested that neoadjuvant therapy and postoperative IM adjuvant therapy improved DFS.

**Conclusion:**

Rectal GISTs are relatively rare and highly malignant tumors. Postoperative oral IM therapy can improve the DFS and OS of intermediate and high risk patients. In patients with rectal GISTs with diameters > 5 cm, IM neoadjuvant therapy can improve anal retention rate, preserve the structure and function of the organs, reduce enterostomy rate, and improve prognosis.

## Introduction

Rectal gastrointestinal stromal tumors (GISTs) account for 3–5% of all GISTs, less than those of the stomach and small intestine [1–3]. The onset of rectal GIST is rare and insidious, and it is anatomically located in the narrow pelvis, which is close to important structures such as the reproductive and urinary systems. In addition, the tumor was close to the dentate line, and the operation may have damaged the anal sphincter. These factors make anal preservation challenging [4, 5]. Currently, the clinicopathological features and treatment methods for rectal GISTs, especially large sample sizes of IM neoadjuvant therapy of rectal GIST patients, have seldom been reported domestically and abroad. In the present study, data were obtained from 101 patients with rectal GISTs who received treatment at the West China Hospital of Sichuan University and the Affiliated Hospital of Guizhou Medical University. This study may help in understanding the clinicopathological characteristics and prognosis of rectal GISTs, especially the significance of IM neoadjuvant therapy.

## Patients and methods

### Patient selection

Hospitalization data were obtained, and follow-up was conducted on 101 patients with rectal GISTs who were treated at West China Hospital of Sichuan University and the Affiliated Hospital of Guizhou Medical University from August 2002 to November 2020. The study was approved by the Institutional Review Board of each institution, and all patients provided informed consent for participation. The inclusion criteria were as follows: (1) 18 years ≤ age < 80 years; (2) rectal GISTs were confirmed by postoperative pathology, immunohistochemical examination or genetic testing after surgical resection; (3) distant metastasis was excluded by chest and abdominal CT examination; (4) patients without serious heart, lung, kidney or other complications tolerated targeted therapy; (5) the Eastern Cooperative Oncology Group (ECOG) performance score < 2; and (6) patients with complete clinical data and follow-up. The exclusion criteria were as follows: (1) pregnant or lactating women (age < 18 years); (2) patients with other major systemic diseases or distant metastasis; and (3) patients whose clinical data were incomplete or lost to follow-up.

### Clinical case collection

All data, including age, sex, patient history, clinical symptoms, imaging data, ECOG performance score, type of surgery, surgical data (including operation time, blood loss, postoperative hospital stay, anal retention rate, enterostomy rate), postoperative treatment and prognosis data, were collected retrospectively and analysed. Resected specimens were reviewed by pathologists from each institution, and the risk of recurrence after surgery was classified according to the modified National Institutes of Health (NIH) criteria [6].

### Therapeutic method

Patients received direct surgery: After the operation, patients were classified into very low, low, intermediate and high risk according to the modified NIH criteria. Periodic follow-up is needed for very low and low risk patients. IM was orally administered for at least 1 year for intermediate risk patients and at least 3 years for high risk patients after surgery.

Patients received IM neoadjuvant therapy: Patients were administered 400 mg/day IM tablets preoperatively for at least 3 months. The patients needed check-up every 3 months. The evaluation was performed according to Choi criteria for the efficacy evaluation of modified solid tumours [7]. Those with a favourable response after neoadjuvant therapy underwent surgery. A dose of 400–600 mg/day IM was suggested orally after surgery according to the genetic results.

### Follow up

A total of 101 patients were followed up until March 31, 2021, and the median follow-up time was 65.7 months (6–240 months). After surgery, patients entered regular outpatient follow-up using a combination of blood tests, rectal palpation, colonoscopy and imaging evaluation at determined intervals. Disease-free survival (DFS) was defined as the time from the date of surgery to the time of recurrence or death due to disease progression, while overall survival (OS) was defined as the time from the date of surgery to the last follow-up or death. Patients with intermediate and high risk were followed up every 3 months in the first 3 years, every 6 months in the following 2 years, and then annually thereafter. Very low or low risk patients were followed up every 6 months for the first 5 years and annually thereafter. Follow-up was stopped when patients died.

### Statistical analysis

SPSS 22.0 statistical software was used to analyse the data. Measurement data are expressed as the mean ± standard deviation (*x* ± *s*), and a t test was used. Enumerative data are expressed as absolute numbers and percentages and were compared by *χ*^*2*^ test or Fisher’s exact probability method. Kaplan–Meier survival analysis was used to draw the survival curve (log-rank was used for the difference test). Cox survival regression was used for survival regression analysis, and P < 0.05 was considered statistically significant.

## Results

A total of 3316 GIST patients, including 138 rectal GISTs, were enrolled in the two hospitals. Excluding 37 patients who were lost to follow-up, had distant metastasis or had incomplete data, 101 patients with rectal GISTs were included in this study, and the clinicopathological characteristics and treatment method information are shown in detail in Table 1. Of the 101 patients, 35 patients experienced recurrence or metastasis, 19 of the 35 patients had isolated local recurrence, 9 had liver metastasis, 7 had abdominal dissemination, and the median time was 33.9 ± 21.1 months (5–96 months). Twenty-eight of them died, and the median time was 52.7 ± 21.9 months (6–108 months).

**Table 1:** The clinicopathological characteristics, surgical data of IM neoadjuvant therapy and direct surgery for all 101 rectal GIST patients

Variables	IM neoadjuvant therapy (*N* = 31)	Direct surgery (*N* = 70)	Number (*N* = 101) (*N*/%)
Age^a^ (years)			
≤ 60	17	45	62 (61.4)
> 60	14	25	39 (38.6)
Sex			
Male	17	47	64 (63.4)
Female	14	23	37 (36.6)
Different era			
2002–2011	0	22	22 (21.8)
2012–2020	31	48	79 (78.2)
ECOG performance score			
0	29	62	91 (90.1)
1	2	8	10 (9.9)
Initial clinical manifestation			
Rectal bleeding	10	10	20 (19.8)
Change in bowel of stool	4	11	15 (14.9)
Change in bowel habit	5	13	18 (17.8)
Rectal discomfort	5	12	17 (16.8)
Digital rectal examination^b^	3	15	18 (17.8)
Others	4	9	13 (12.9)
Tumor size^c^ (cm)			
≤ 5	2	34	36 (35.6)
> 5	29	36	65 (64.4)
Approach of surgery			
Laparotomy	25	57	82 (81.2)
Laparoscopy	6	8	14 (13.9)
Endoscopy	0	5	5 (4.9)
Type of surgery			
Local	30	65	95 (94.1)
Radical	1	5	6 (5.9)
Procedure of surgery^d^			
LAR	27	43	70 (69.3)
ISR	2	7	9 (9.0)
APR	2	15	17 (16.8)
LR	0	5	5 (4.9)
Covering stoma in sphincter-preserving surgery (LAR + ISR)^e^			
Yes	3	10	13 (16.5)
No	26	40	66 (83.5)
Surgical margin			
Positive	0	4	4 (4.0)
Negative	31	66	97 (96.0)
Tumor rupture			
Yes	0	0	0 (0.0)
No	31	70	101 (100.0)
Postoperative complications			
Rectal bleeding	0	1	1 (1.0)
Anastomotic fistula	1	4	5 (4.9)
Wound infection	2	4	6 (5.9)
Abdominal infection	1	2	3 (3.0)
Others	1	4	5 (4.9)
None	26	55	81 (80.3)
Mitotic index (50HPF)			
≤ 5	22	28	50 (49.5)
> 5	9	42	51 (50.5)
Pathological feature			
Spindle	28	54	82 (81.2)
Epithelial	2	13	15 (14.9)
Mixed	1	3	4 (3.9)
Immunohistochemistry			
CD117 positive	29	69	98 (97.0)
DOG-1positive^f^	29	46	75 (96.2)
CD34 positive	28	55	83 (82.1)
Modified NIH criteria^g^			
Very low risk	–	8	8 (11.4)
Low risk	–	10	10 (14.3)
Intermediate risk	–	7	7 (10.0)
High risk	–	45	45 (64.3)
Genetic mutation^h^			
KIT Exon 11 mutation	14	25	39 (83.0)
KIT Exon 9 mutation	2	2	4 (8.5)
Wild type mutation	1	3	4 (8.5)
Postoperative IM adjuvant treatment			
Yes	25	24	49 (48.5)
No	6	46	52 (51.5)

^a^Age: 22–79 years (55.4 ± 12.2 years)

^b^17.8% of the patients were found initially during the rectal palpation, and a total of 69.3% (70/101) of all the patients could be found by this examination

^c^The median size was 6.18 ± 3.02 cm

^d^LAR: Low anterior resection; ISR: Intersphincteric resection; APR: Abdominoperineal resection; LR: local resection [8]

^e^A total of 79 patients had sphincter-preserving surgery (LAR + ISR)

^f^Only 78 patients underwent DOG-1 testing

^g^Excluding 31 neoadjuvant patients who were not applicable to modified NIH criteria,70 patients left

^h^Only 47 patients underwent genetic testing

### Analysis of patients receiving direct surgery and neoadjuvant therapy

#### Direct surgery

**Fig. 1:**
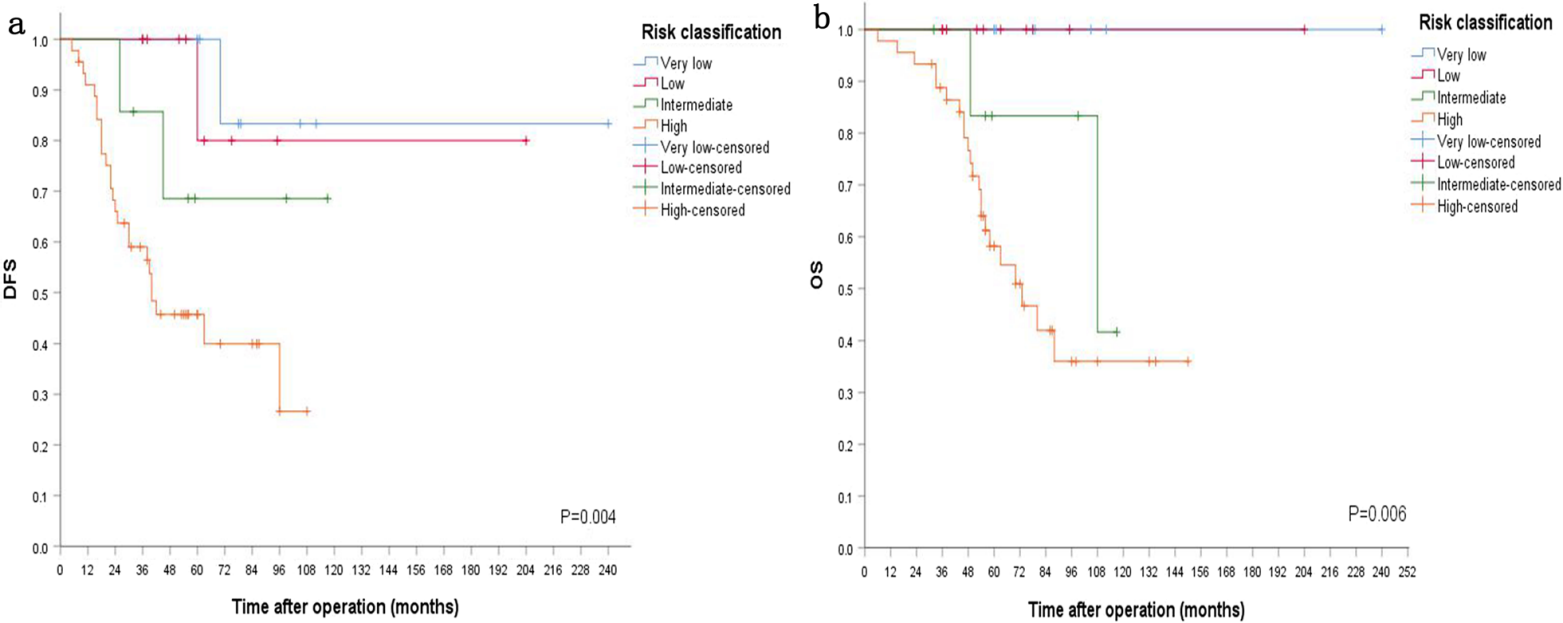
Prognosis of 70 patients who received direct surgery. **a** DFS stratified by modified NIH risk classification. **b** OS stratified by modified NIH risk classification

Seventy patients with a tumor size of 0.8–19.3 cm (5.63 ± 3.28 cm) underwent direct surgery, which included 8 very low risk, 10 low risk, 7 intermediate risk, and 45 high risk patients. Twenty-nine patients recurred or metastasized, including 1very low risk, 1 low risk, 2 intermediate risk and 25 high risk patients. The time of recurrence was 30.7 ± 20.6 months (5–96 months). Twenty-four of them died because of disease progression, including 2 intermediate risk and 22 high risk patients. The time of death was 51.5 ± 22.4 months (6–108 months). Cox regression analysis was performed on sex, age, era, diameter, invasiveness, ECOG performance score, approach of surgery, type of surgery, surgical margin, mitotic index, risk classification, and postoperative IM adjuvant therapy, and the results showed that risk classification (P = 0.030) and postoperative IM adjuvant therapy (P = 0.002) affected DFS. Meanwhile, risk classification (P = 0.021) and postoperative IM adjuvant therapy (P = 0.041) were also related to OS. Kaplan–Meier survival analysis of 70 patients undergoing direct surgery suggested that both risk classification and postoperative adjuvant therapy affected DFS and OS. Kaplan–Meier risk classification is shown in Fig. 1.

**Fig.2:**
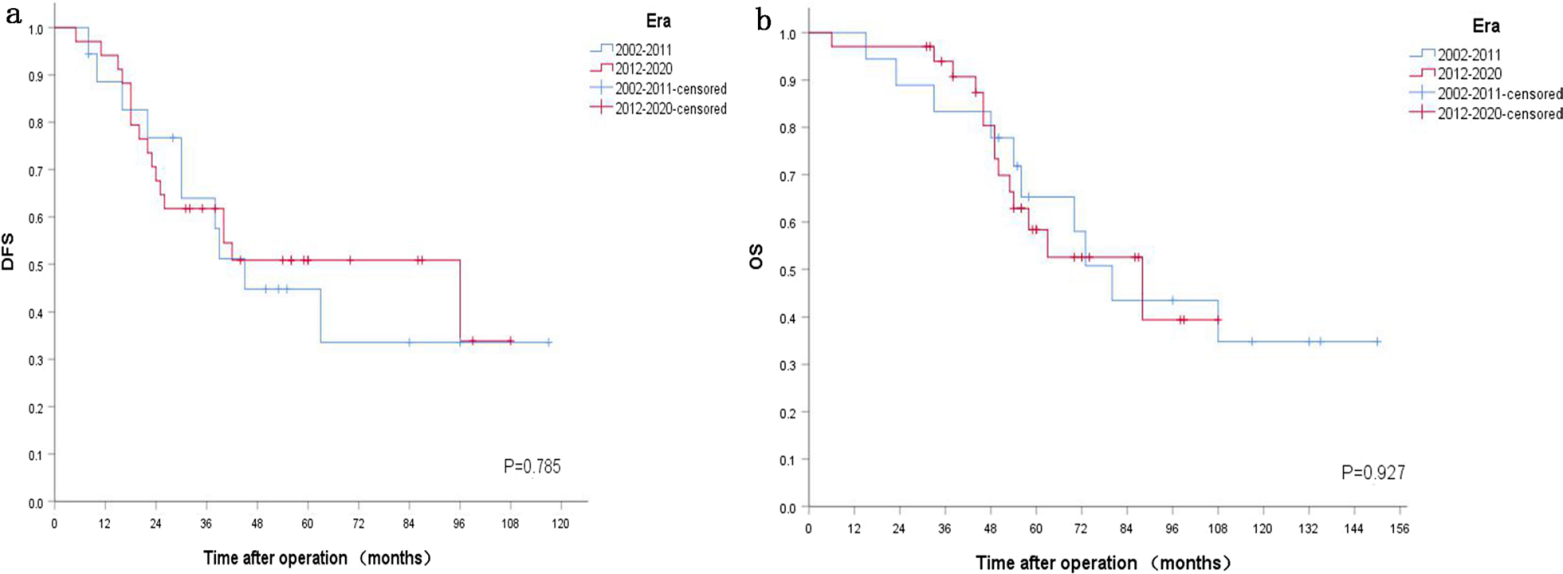
Prognosis of 52 patients with intermediate and high risk receiving direct surgery. **a** DFS stratified by different era. **b** OS stratified by different era

**Fig. 3:**
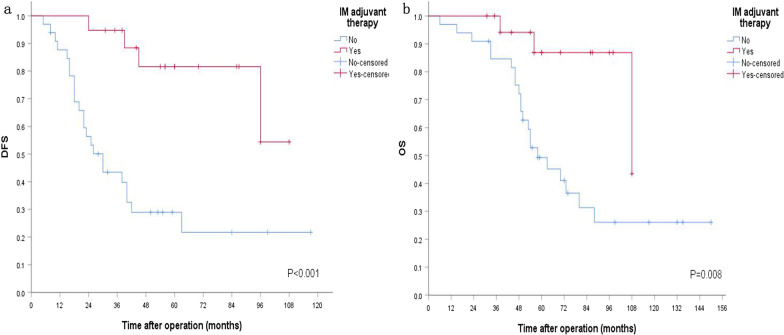
Prognosis of 52 patients with intermediate and high risk receiving direct surgery. **a** DFS stratified by postoperative IM adjuvant treatment. **b** OS stratified by postoperative IM adjuvant treatment

Excluding 18 patients with very low risk and low risk patients, 52 intermediate and high risk patients left. Cox regression analysis showed that sex (P = 0.173), age (P = 0.354), era (P = 0.702), diameter (P = 0.877), invasiveness (P = 0.247), ECOG Performance Score (P = 0.211), approach of surgery (P = 0.467), type of surgery (P = 0.195), surgical margin (P = 0.682), mitotic index (P = 0.921), risk classification (P = 0.067) had no differences of DFS, but post-operative IM adjuvant treatment improved their DFS (P = 0.003). Meanwhile, sex (P = 0.084), age (P = 0.473), era (P = 0.487), diameter (P = 0.908), invasiveness (P = 0.802), ECOG Performance Score (P = 0.677), approach of surgery (P = 0.143), type of surgery (P = 0.230), surgical margin (P = 0.169), mitotic index (P = 0.593), risk classification (P = 0.235) did not affect OS, however, post-operative IM adjuvant treatment could improve their OS, too (P = 0.032). Kaplan–Meier survival analysis showed that there were no era differences of DFS and OS (P = 0.785, P = 0.927, respectively) (Fig. 2). However, Kaplan–Meier survival analysis revealed IM adjuvant treatment improved DFS and OS (P < 0.001, P = 0.008, respectively) (Fig. 3).

#### IM neoadjuvant therapy

**Fig. 4:**
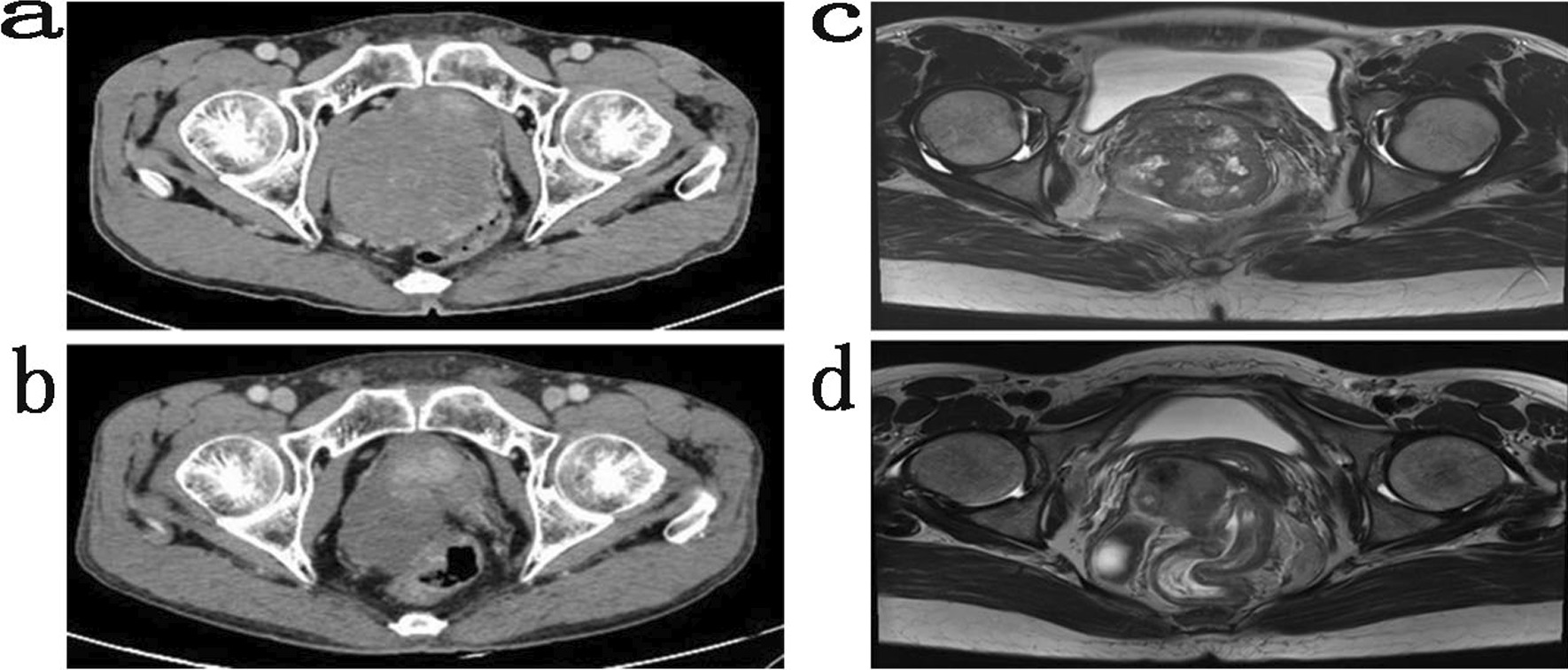
Imaging comparison of two patients before and after IM neoadjuvant treatment. Enhanced CT scan of the rectal GIST patient who received IM neoadjuvant therapy for 6 months (**a** initial tumor, and **b** tumor after 6 months of IM neoadjuvant therapy). Magnetic resonance imaging of another rectal GIST patient treated with IM neoadjuvant therapy for 9 months (**c** initial tumor, and **d** tumor after 9 months IM neoadjuvant therapy)

Thirty-one patients received IM neoadjuvant therapy for 3–15 months (6.1 ± 2.5 months): complete response (CR) 0, partial response (PR) 26, stable disease (SD) 4 and progressive disease (PD) 1. The objective response rate (ORR) was 83.9% (26/31), and the disease control rate (DCR) reached 96.8% (30/31). The diameter before treatment was 3.60–11.90 cm (7.23 ± 1.90 cm), and neoadjuvant therapy lasted 3–15 months (6.1 ± 2.5 months). After treatment, the diameter was 1.70–9.50 cm (4.77 ± 1.67 cm), but the difference was not statistically significant (P = 0.465). The images of some patients before and after IM neoadjuvant therapy are shown in Fig. 4. During the follow-up, six patients experienced recurrence, the time to recurrence was 49.5 ± 16.9 months (26–69 months); And 4 of them died, the time of death was 59.5 ± 19.7 months (33–78 months).

### Subgroup analysis of diameter

**Fig. 5:**
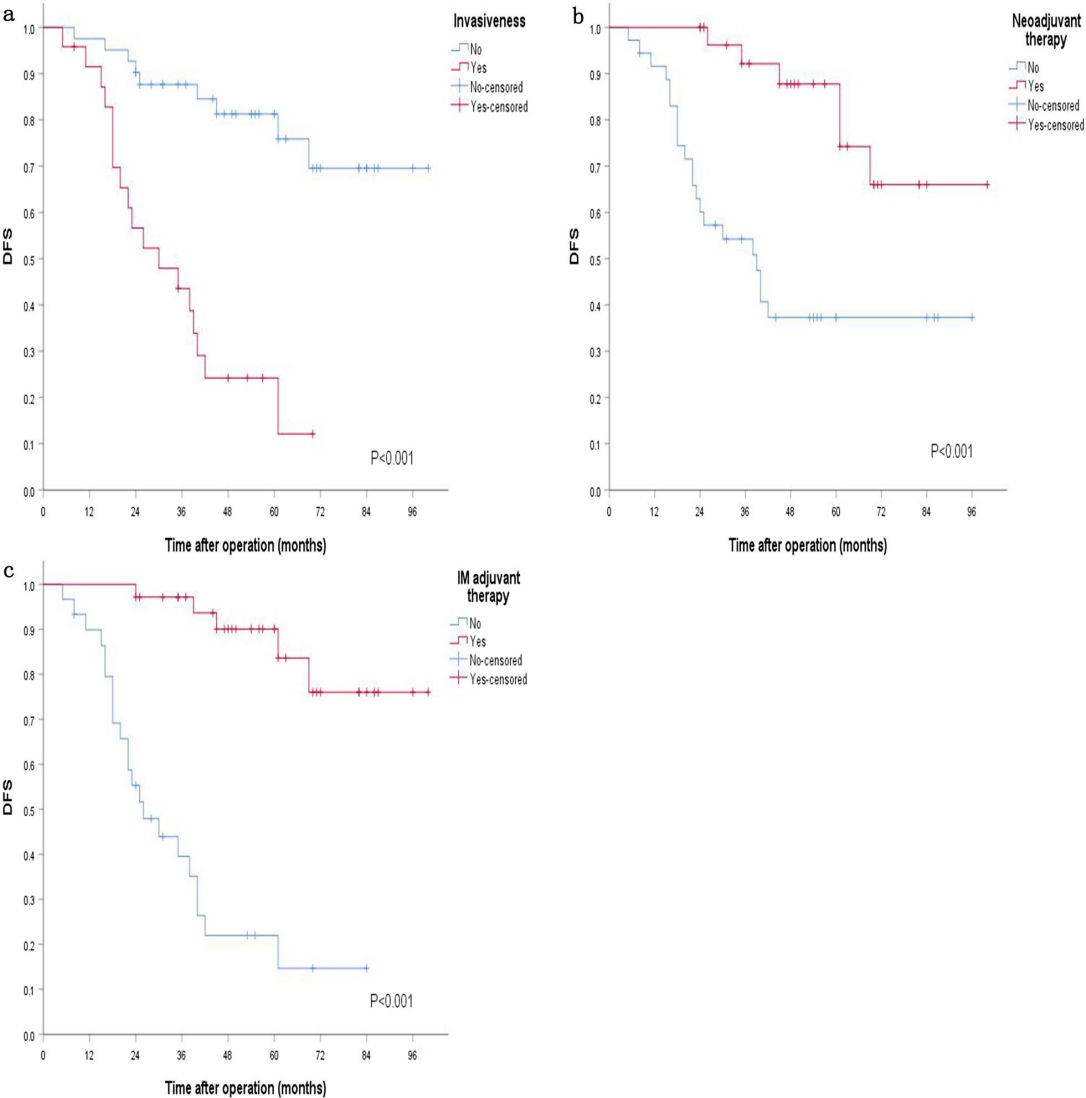
DFS of patients with a diameter > 5 cm stratified by invasiveness, neoadjuvant therapy and postoperative therapy. **a** DFS stratified by invasiveness. **b** DFS stratified by IM neoadjuvant therapy. **c** DFS stratified by postoperative IM adjuvant therapy

1. Among 36 patients with a tumor diameter ≤ 5 cm, two patients received IM neoadjuvant therapy, and 34 patients received direct surgery. No factors (sex, age, ECOG performance score, invasion, approach of surgery, type of surgery, surgical margin, mitotic index, IM neoadjuvant therapy and IM adjuvant therapy) affecting DFS or OS were found in univariate analysis or Cox regression analysis.
2. Among the 65 patients with a tumor diameter > 5 cm, 29 patients received IM neoadjuvant therapy, and 36 patients received direct surgery. No significant difference was observed in operative time (P = 0.621) or recovery time of gastrointestinal function (P = 0.222) between the two groups. However, the neoadjuvant treatment group was superior to the direct surgery group in terms of blood loss (P = 0.022) and postoperative hospital stay (P = 0.001), the anal retention rate increased (P = 0.031), and the stoma rate decreased (P = 0.037). Details are shown in Table 2. Ka-square univariate analysis indicated that invasiveness of the surrounding organs (P < 0.001), neoadjuvant therapy (P = 0.002), type of surgery (P = 0.022), mitotic index (P = 0.001), and adjuvant treatment of IM after surgery (P < 0.001) affected DFS, while sex (P = 0.918), age (P = 0.437), ECOG performance score (P = 0.367), approach of surgery (P = 0.741) and surgical margin (P = 0.133) did not affect DFS. Among the above ten factors, Cox regression analysis indicated that invasiveness of the surrounding organs (P = 0.040), neoadjuvant treatment (P = 0.028) and adjuvant imatinib (P = 0.003) affected DFS, and the other seven factors had no effect. Meanwhile, none of the above ten factors were found to affect OS. Kaplan–Meier survival analysis suggested that invasion of the surrounding organs (P < 0.001), neoadjuvant therapy (P < 0.001) and postoperative IM adjuvant therapy (P < 0.001) affected recurrence and metastasis, as shown in Fig. 5. Remove deleted patients, the 3-year DFS, 3-year OS, 5-year DFS, 5-year OS of the patients who received IM neoadjuvant therapy and those who received direct surgery were (23/25) 92% vs. (18/36) 50%, P = 0.001; (24/25) 92% vs. (32/36) 88.9%, P = 0.602; (13/16) 81.3% vs. (10/32) 31.3%, P = 0.001; (13/16) 81.3% vs. (16/32) 50%, P = 0.007.

**Table 2:** Comparison of surgical conditions of 65 patients with diameters > 5 cm

Group	Number	Operation time (min)	Bleeding amount (ml)	Recovery time of gastrointestinal function (day)	Postoperative hospital stay (day)	Anal retention rate	Enterostomy rate
Neoadjuvant group	29	120.0 ± 35.5	91.2 ± 17.7	2.9 ± 0.9	7.3 ± 2.3	27/29 (93.1%)	3/29 (10.3%)
Direct Surgery	36	154.6 ± 38.0	226.7 ± 22.3	3.7 ± 1.2	10.3 ± 4.9	26/36 (72.2%)	12/36 (33.3%)
P value		0.621	0.022	0.222	0.001	0.031	0.037

## Discussion

Rectal GISTs are relatively rare, accounting for about 3–5% of GISTs. Our study showed that rectal GISTs accounted for 4.2% of all GISTs (138/3316), similar to the Rebecca report [9]. The median size of rectal GISTs was 5 cm [8, 10], but the median size obtained in our study was 6.18 ± 3.02 cm, which was slightly larger than that of the above studies. The clinical symptoms of rectal GISTs are related to the size of the tumor. When the tumor is less than 2 cm, no clinical symptoms are observed, and they are often found in the physical examination. However, as the tumor gradually grows into the intestinal cavity, a series of symptoms occur, including stool trait changes, defecation habit changes, anal discomfort or tumor rupture caused by blood in the stool, and late invasion of the surrounding organs can also manifest as hemuria and vaginal bleeding [11]. Our study showed that rectal bleeding, changes in the bowel of stool, changes in bowel habits and anal discomfort were 19.8%, 14.9%, 17.8% and 16.8%, respectively. Therefore, its clinical manifestations have no specific manifestations compared to other rectal diseases. However, rectal GISTs tend to occur in the middle and lower segments [8, 9]. It is worth mentioning that 17.8% of our patients did not have any symptoms but were found to initiate rectal palpation, and 69.3% (70/101) of all the patients could be found by rectal palpation, similar to the studies, Therefore, rectal palpation plays an important role in the early discovery and differential diagnosis of rectal GISTs.

The pathological diagnosis of rectal GISTs is mainly based on histological and immunohistochemical results [12]. The cell morphology was divided into spindle, epithelioid, and mixed cell types, which accounted for 97.0%, 96.2% and 82.1% in our study, respectively, similar to GISTs in other sites. In terms of immunohistochemistry, CD117 and DOG-1 had the most diagnostic value, and CD34 was very significant for its diagnosis. Miettinen et al. reported that the expression rates of CD117 and CD34 in 96 cases of rectal mesenchymal tumours were 100% and 94%, respectively [13]. In our study, the positive rates of CD117 and DOG-1 were 97.0% (98/101) and 96.2% (75/78), respectively, and CD34 also reached 82.1% (83/101); thus, CD117 was similar to Miettinen’s report, but CD34 was slightly lower. According to Miettinen’s report, KIT exon 11 mutations are common in rectal GISTs, followed by exon 9 mutations and wild-type mutations, but PDGFRA mutations are rare in rectal GISTs. Similar results were obtained in the present study, where KIT exon 11 (38/47), KIT exon 9 (4/47) and wild-type (4/47) were detected; however, no PDGFRA mutation patients were found in our study. Rectal GISTs often have a layer of pseudocapsule on the surface, rarely infiltrate along the intestinal wall, and rarely have lymph node metastasis. Hence, lymph node dissection is not necessary [14]. Among the 101 patients in the present study, 76 lymph nodes were dissected, but no metastasis occurred, thus confirming the above view.

Rectal GIST is a disease with a high recurrence rate. Surgical resection is still the most important treatment. The surgical principle is to complete excision, maintain the integrity of the capsule, and avoid rupture. The malignant risk of rectal GIST is higher than that of stomach GIST and is closer to that of intestinal GIST. Yasui et al. reported that the proportion of rectal high-risk GISTs was 45%, while the MSKCC single centre reported 72.3% [4, 15]. For the 70 patients who underwent direct surgery, 45 cases were at high risk after surgery, accounting for 64.3%, which was higher than the result obtained by Yasui. However, considering that 31 patients with large diameters could not be evaluated by modified NIH criteria after IM neoadjuvant therapy, our ratio of high risk would be higher than this value, supporting the MSKCC data. Cox regression analysis was performed on sex, age, era, diameter, invasiveness, ECOG performance score, approach of surgery, type of surgery, surgical margin, mitotic index, risk classification, and postoperative IM adjuvant therapy of seventy patients. The results showed that risk classification and postoperative IM adjuvant therapy affected DFS and OS. Therefore, for patients with intermediate and high risk rectal GISTs, postoperative adjuvant treatment with imatinib is particularly important for improving their prognosis.

For patients with large tumors prone to intraoperative bleeding and tumors close to the anal margin, IM neoadjuvant therapy can be considered, and this treatment will result in an obvious tumor descent effect, improve the anal preservation rate, reduce the positive rate of the surgical margin, and improve the prognosis of patients [16–19]. At present, the time of IM neoadjuvant therapy is appropriate within 6–12 months according to NCCN and ESMO guidelines [16, 17]. Each guideline recommends that the duration of neoadjuvant treatment be defined as the maximum response to medication. The maximum response time was defined as two consecutive enhanced CT or MRI scans indicating no remission of the tumor. At this time, surgical resection should be performed immediately given an opportunity for surgery [20]. With prolonged drug treatment time, secondary mutations may occur during treatment. Bednarski et al., in a retrospective study of 93 patients treated preoperatively, showed that a neoadjuvant treatment time of > 365 days was associated with an increased progression rate [21]. Therefore, blindly prolonging the IM neoadjuvant treatment time to maximize is highly likely to lead to drug resistance and then miss the best operative timing [22]. In our present study, 31 patients were treated for 3–15 months (6.1 ± 2.5 months). During IM neoadjuvant treatment, the patients were generally followed up and evaluated dynamically every 3 months in a timely manner to understand the effect of neoadjuvant therapy and accurately determine the timing of surgery. In the present study, the ORR was 83.9%, and the DCR reached as high as 96.8%. This result was similar to the Kanedo report involving 6 retrospective studies in 118 patients with neoadjuvant rectal GISTs with a response rate of 70.3% and a control rate of 99.2% [23].

Considering that rectal GISTs are located in a narrow pelvis with a special anatomical structure and adjacent to important structures such as the reproductive and urinary systems, the diameter of the tumor is the most important indicator for us to consider IM neoadjuvant therapy. Thus, we conducted a subgroup analysis of the diameter and found that no factors, including IM neoadjuvant therapy, affected DFS and OS in patients with a diameter of ≤ 5 cm. However, considering the small sample size, whether IM neoadjuvant therapy in patients with a diameter of ≤ 5 cm can provide a survival benefit for rectal GISTs needs to be further verified. However, for patients with tumor diameters > 5 cm, univariate analysis (P = 0.002) and multivariate analysis (P = 0.028) both indicated that neoadjuvant therapy can improve DFS. Therefore, it was the same as Vallilas’s report that IM neoadjuvant therapy for specific sites or large tumors can improve the prognosis [15]. Meanwhile, it is worth mentioning that in our study, whether the surgical margin was positive did not affect the prognosis, and there was no need for further surgical resection, which was consistent with Cavnar’s and Gronchi’s report [24, 25]. In summary, for patients with a diameter ≤ 5 cm, considering that the composition of IM neoadjuvant therapy was relatively low, with only 2 cases, the data analysis might be biased. Whether neoadjuvant therapy in patients with a diameter ≤ 5 cm can provide a survival benefit for rectal GISTs needs to be further verified in a multicentre, large-sample prospective study. However, for rectal GISTs with diameters > 5 cm, our univariate and Cox regression analyses both showed that neoadjuvant treatment could improve the prognosis of patients. It could improve 3-year RFS and 5-year RFS and OS. Therefore, for rectal GIST patients with a diameter of > 5 cm, we recommend IM neoadjuvant therapy and then surgery to descend the tumor and improve the prognosis.

Moreover, from subgroup analysis of the diameter, our research showed that neoadjuvant therapy with a diameter > 5 cm could improve the safety of the surgery, preserve the anus, decrease the possibility of enterostomy and improve the postoperative quality of life of patients. At present, how to shrink tumors that are difficult to completely resect and how to improve the anal preservation rate of patients with rectal GISTs have been widely studied [26]. It has been reported that IM neoadjuvant therapy can reduce bleeding and improve the safety of surgery, which may be attributed to the reduction in tumor volume and fibrosis, hyaline degeneration, and toughening of tumor texture caused by drugs, making it less prone to rupture and bleeding during surgery. Our study also confirmed the value of neoadjuvant therapy with a shorter postoperative hospital stay (P = 0.001) and less bleeding (P = 0.022). Moreover, neoadjuvant therapy can significantly reduce the tumor diameter, and this condition is conducive to the implementation of organ preservation surgery [27, 28]. The MSKCC single-centre study showed that IM neoadjuvant adjuvant therapy significantly increased the anal retention rate (92% vs. 48%). Our study showed that neoadjuvant therapy could increase the anal retention rate (93.1% vs. 72.2%, P = 0.031) and reduce the rate of enterostomy (10.3% vs. 33.3%, P = 0.037), demonstrating the value of IM neoadjuvant therapy in preserving organ function.

In conclusion, rectal GIST is a disease with a special location, high malignancy and high recurrence rate. Postoperative IM adjuvant treatment can reduce the recurrence and metastasis rate of intermediate and high risk patients. IM neoadjuvant therapy can reduce tumor volume, protect organ structure and function, and improve the prognosis of patients with a diameter > 5 cm. However, the neoadjuvant treatment of rectal GISTs remains lacking domestically and abroad. In the future, more prospective multicentre studies are needed to further explore and draw conclusions.

## Data Availability

The datasets used and analysed during the current study are available from the corresponding author on reasonable request.

## Abbreviations

GISTs: Gastrointestinal stromal tumors
IM: Imatinib mesylate
DFS: Disease-free survival (DFS)
OS: Overall survival
NIH: National Institutes of Health
CR: Complete response
PR: Partial response
SD: Stable disease
PD: Progressive disease
ORR: Objective response rate
DCR: Disease control rate
ECOG: Eastern Cooperative Oncology Group
LAR: Low anterior resection
ISR: Intersphincteric resection
APR: Abdominoperineal resection
LR: Local resection
